# Can digital transformation enhance labor productivity in enterprises: An analysis from the perspective of business process reengineering

**DOI:** 10.1371/journal.pone.0325484

**Published:** 2025-06-26

**Authors:** Yi Zhou, Jialun Lyu, Li Li

**Affiliations:** 1 School of Economics, Beijing Technology and Business University, Beijing, Beijing, China; 2 Capital Circulation Industry Research Base, Beijing, Beijing, China; Southwestern University of Finance and Economics, CHINA

## Abstract

From the perspective of business process reengineering, this paper analyzes the impact of digital transformation on labor productivity in enterprises and its underlying mechanisms. The study finds that digital transformation significantly enhances labor productivity in enterprises, with both the application of digital technologies and innovation in digital technology scenarios having a notable positive effect. Furthermore, digital transformation improves labor productivity mainly by optimizing production management processes, reducing human resource redundancy, enhancing the efficiency of human resource utilization, and improving internal control mechanisms to enhance decision-making efficiency. The effects of digital transformation on labor productivity are more pronounced in non-state-owned enterprises and enterprises that are highly dependent on their industrial chains. Further analysis shows that the level of industry monopoly and the overall digitalization level of the industry play a moderating role in the process by which digital transformation affects labor productivity.

## Introduction

The “14th Five-Year Plan for National Economic and Social Development” (2021–2025) clearly proposes, “To enhance the quality and efficiency of economic development, ensuring that the growth rate of overall labor productivity surpasses that of GDP.” However, in the process of promoting high-quality development of the real economy, China faces the dilemma of low and relatively slow growth in labor productivity. According to the World Bank data, as of 2023, China’s labor productivity, calculated based on purchasing power parity (PPP), is only 27.7% of that of the United States and 35.4% of the average of high-income countries. Notably, with the gradual decline in labor supply, the traditional economic growth model that relies on the demographic dividend and urbanization is becoming increasingly unsustainable. Therefore, improving labor productivity in enterprises has become a key driver of sustained high-quality economic development. The report of the 20th National Congress of the Communist Party of China further emphasizes that the construction of a modern industrial system requires focusing economic development on the real economy, continuously promoting the deep integration of the digital economy with the real economy, and resolutely advancing the construction of new industrialization. In a congratulatory message to the 2023 China International Smart Industry Expo, President Xi Jinping reiterated the need to “advance both digital industrialization and industrial digitalization, accelerate the construction of a network power and a digital China”.

In fact, digital technologies not only optimize production processes to improve production efficiency but also enable precise decision-making and personalized services to expand markets, thereby enhancing the competitiveness of the real economy. It is clear that the integration of digital and real economies (“digital-real integration”) will be a crucial means to improve labor productivity in China and achieve high-quality economic development. China has made significant achievements in digital technology innovation and digital infrastructure construction, laying a solid foundation for the deep integration of the digital economy and the real economy. Among them, digital transformation is a key method for digital-real integration, and under this context, digital transformation has already become an important pathway for enterprises to improve labor productivity.

However, it is worth noting that according to data from the National Bureau of Statistics, the growth rate of labor productivity in China has shown a downward trend since 2007. Additionally, the Solow Paradox raises doubts about whether information and communication technology (ICT) can effectively enhance productivity. Scholars like Acemoglu and Brynjolfsson also suggest that the application of digital technologies may disrupt employment opportunities and wage levels, potentially slowing down overall economic efficiency [1,2]. This naturally raises the question: can the embedding of digital technologies effectively enhance labor productivity in the real economy, and through what mechanisms? Existing research on the functionality of digital transformation mainly focuses on three aspects: first, the belief that digital transformation enables enterprises to expand revenue sources by creating new business models and breaking traditional market constraints [3]; second, the view that digital technology promotes enterprise R&D collaboration, optimizes product development processes, and reduces innovation costs to enhance enterprise innovation capabilities [4]; third, the understanding that digital tools improve supply chain management and increase business process automation, thus enhancing operational efficiency and, consequently, total factor productivity [5]. Business Process Reengineering (BPR) refers to the fundamental redesign and systematic optimization of existing business processes through digital technologies and other means, aiming to eliminate redundant steps, improve resource utilization efficiency, enhance collaboration capabilities, and ultimately achieve the reconstruction of the production function and an increase in labor productivity. The impact of digitalization on a company’s business processes and development model is clearly reflected at the level of labor productivity. More importantly, digital transformation will also directly influence labor productivity through capital deepening. However, unfortunately, existing research has not further explored the relationship between digital transformation and corporate labor productivity.

Therefore, this paper aims to delve deeper into the impact of digital technology integration on labor productivity in the real economy and its theoretical mechanisms, including how digital transformation influences labor productivity by optimizing business processes, as well as the role of industry characteristics in digital transformation’s effect on labor productivity. The marginal contributions of this paper are as follows: First, existing literature tends to focus more on the impact of enterprise digital transformation on total factor productivity, with less attention paid to its impact on the efficiency of human capital utilization. This paper further explores how digital transformation affects labor productivity from the perspective of business process reengineering, thus enriching the literature on the economic consequences of enterprise digital transformation. Second, this paper introduces the theory of lean production and research on internal control systems to investigate the theoretical mechanisms through which digital transformation influences labor productivity, offering insights into the field of human resource management theory. The conclusions of this study have practical implications for human capital allocation in enterprises.

## 1. Theoretical analysis

### 1.1 Digital transformation and labor productivity

Digital transformation facilitates enterprises in breaking organizational inertia by reconfiguring the capital-labor ratio to optimize production functions, thereby enhancing labor productivity. This is primarily reflected in three aspects: First, automation and digital tools improve employee efficiency, allowing them to focus on tasks with higher cognitive value [6]. For instance, the introduction of industrial robots and other production technologies takes over many repetitive, low-value tasks, while artificial intelligence and other digital technologies empower enterprises to achieve breakthroughs in data analysis, decision support, predictive maintenance, and personalized services. The introduction of these digital production technologies enables employees to concentrate more on innovation, strategic planning, and complex problem-solving, ultimately enhancing overall production efficiency and competitiveness.

Second, the various mobile technologies and digital hardware devices introduced during the digital transformation process provide favorable conditions for digital learning within enterprises. This encourages organizations to integrate social learning potential, incorporating social elements such as knowledge sharing, informal problem-solving, and user-generated content into content design and delivery [7]. These practices further enhance employees’ digital learning capabilities, promote knowledge management, and improve team collaboration, creating a more cooperative and agile work environment [8]. Knowledge sharing and collaboration among employees not only increase the overall intellectual capital of the team but also help reduce redundant labor and accelerate the training and integration of new employees [9].

Finally, the accumulation and application of digital capabilities within enterprises, including data collection, analysis, and decision-making, improve management efficiency, eliminate internal “data silos,” and enhance the organization and allocation efficiency of human resources. Based on this, we propose the following research hypothesis:

#### Hypothesis 1.

Digital transformation in enterprises contributes to the enhancement of labor productivity.

### 1.2 Digital transformation, process reengineering, and labor productivity enhancement

From the resource-based view (RBV), the key to digital transformation lies in utilizing Information and Communication Technology (ICT) to establish a comprehensive information system that enhances the strategic value of enterprises. In the 1970s, management information systems (MIS) and strategic information systems (SIS) based on ICT emerged. Early MIS primarily provided information support for management and decision-making in a mechanical manner through database formats. However, with the emergence and development of big data, artificial intelligence, and other technologies, digital transformation has begun to exert new influences on enterprise business models. The application of digital technologies can provide diversified management solutions for optimizing business processes based on real-world contexts, driving the transformation of business models from “mechanical economy” to “thinking economy.” In other words, digital transformation is often viewed as a crucial means for enterprises to reduce costs and increase efficiency, profoundly impacting the reengineering of business processes such as production and factor allocation.

Specifically, digital transformation exerts a reengineering effect on business processes by creatively destroying existing workflows. First, enterprises leverage the data analysis capabilities of digital technologies to reorganize business processes [10], thereby influencing their business models and achieving production automation while reducing the complexity of business operations [11]. At this stage, digital technologies influence not only the technical context but also the enterprise’s strategy, management, and overall capabilities [12]. Second, general business processes increasingly emphasize the participation of digital technologies [13], with the integration of digital technologies into business processes evolving beyond isolated instances, leading to the establishment of business processes centered around digital technologies.

Furthermore, business process reengineering can be divided into two parts: the optimization of primary processes directly related to production (production processes) and the improvement of supporting processes necessary for achieving enterprise efficiency, primarily focused on management and decision-making [14]. The optimization of production processes refers to the process of converting labor and other production factors into products or services, which is a core component of upgrading the enterprise value chain. In contrast, supporting non-production processes, such as management, finance, and human resources, primarily ensures efficient enterprise operations by reducing transaction costs and enhancing internal coordination efficiency through improved internal controls.

#### 1.2.1 Digital transformation, production management optimization, and labor productivity enhancement.

The integration of the digital and physical realms transforms the tools of the real economy from industrial technology to information and intelligent technologies, shifting the production space of workers from physical workshops to platforms like the Internet and the Internet of Things [15]. From a production management perspective, this digital transformation helps enterprises overcome outdated production models, ensuring continuous self-optimization of production processes [16]. For instance, in traditional automotive manufacturing, digital transformation has replaced manual welding stations with IoT-connected robotic arms that automatically adjust operations based on real-time quality monitoring data. Specifically, enterprises leverage digital technologies to collect extensive data on the operation of production equipment and products. Through data analysis, enterprises can better monitor production, intervene in shutdowns, alter production methods, and enhance production flexibility to better align with demand-side needs, thereby laying the foundation for production process optimization [17] Meanwhile, according to lean production theory, embedding digital technologies into production processes will also aid in increasing production visibility and transparency, enhancing the enterprise’s lean production capabilities by identifying and optimizing business processes [18], and eliminating various types of production waste such as overproduction, unnecessary transportation, overprocessing, and excessive inventory [19].

Additionally, lean production based on digital technologies contributes to reducing unnecessary labor input and alleviating inefficiencies caused by labor resource mismatches. Specifically, the high standardization, flexibility, and quality brought by lean manufacturing mainly manifest in the improvement of technical efficiency, enabling enterprises to produce more with the same labor input, thus enhancing labor productivity by boosting workers’ production capabilities. Furthermore, digital transformation not only shapes lean production processes and optimizes enterprise production processes but also enhances the efficiency of worker and management participation through increased information transmission and communication intensity [20], thereby improving labor productivity through enhanced collaboration [21].

Based on the above analysis, this paper proposes the following hypothesis:

**Hypothesis 2:** Enterprise digital transformation enhances labor productivity through optimizing production management.

#### 1.2.2 Digital transformation, internal control enhancement, and labor productivity improvement.

Enterprise internal control consists of accounting control and management control. Accounting control refers to the enhancement of financial systems to ensure the accuracy and completeness of accounting information within a reasonable cost range and to assist management in decision-making [22]. In the context of digital transformation, new digital technologies such as big data and social media enrich the means of acquiring accounting information, thereby providing more comprehensive enterprise performance indicators and allowing the accounting process to have a more profound and predictive impact on corporate decisions [23]. Additionally, by leveraging big data and artificial intelligence technologies, managers and financial personnel can directly review all accounting data to identify anomalies and potential risks [24], greatly enhancing the enterprise’s ability to analyze accounting information and making accounting and auditing activities more efficient [25].

Management control refers to coordinating and managing resources and activities through internal management systems to reduce internal transaction costs [26]. On the one hand, resource management based on digital technology is a vital component of internal management control [27], with the functions of knowledge management and dissemination being crucial for improving organizational efficiency. On the other hand, with the aid of emerging digital technologies, information management systems represented by ERP (Enterprise Resource Planning) have gained new vitality, integrating various business processes of the enterprise, providing real-time data and information flow, and reducing information silos and redundant work.

Moreover, the accounting process is essentially regarded as a performance measurement system for the enterprise. A well-developed accounting control system can provide accurate performance evaluation data, set reasonable performance targets and reward mechanisms, and motivate employees to improve work efficiency and output. Goal management theory emphasizes improving employee engagement and work efficiency through clear goals and performance evaluations. For instance, an efficient management control system based on digital technology (an efficient ERP system) can ensure a smoother goal-setting, communication, and evaluation process, reducing unnecessary communication and approval time, and allowing employees to focus on achieving corporate goals [28].

Overall, enterprises can optimize internal management systems through the application of digital technologies, better coordinating and managing internal and external resources, reducing internal transaction costs, and improving organizational synergy and work efficiency. Firstly, digital technologies enable internal control systems to provide more accurate performance evaluation data, set reasonable performance targets and reward mechanisms, motivating employees to enhance work efficiency and output. Secondly, digital technologies improve the accuracy and timeliness of accounting information, allowing management to make better decisions, optimize processes, and adjust plans, thereby improving labor productivity. Thus, digital transformation enhances labor productivity through the improvement of internal control processes. Based on this, the paper proposes the following hypothesis:

**Hypothesis 3:** Enterprise digital transformation enhances labor productivity through improving internal control.

### 1.3 Heterogeneity analysis based on ownership structure

State-owned enterprises (SOEs) and private enterprises differ in organizational structure, decision-making processes, capital allocation, and strategic objectives due to differences in ownership structure, affecting their paths and outcomes in digital transformation. Firstly, SOEs have relatively unique organizational structures and management models, often facing more hierarchical approvals and administrative interventions in decision-making processes, which can lead to slower response times and lower implementation efficiency in digital transformation [29]. In contrast, private enterprises usually have more flexible management mechanisms, allowing them to quickly adapt to market changes and technological innovations, thereby utilizing digital tools more effectively to enhance labor productivity. Secondly, SOEs often exhibit rigidity in resource allocation, with distribution relying on administrative orders rather than market demand, leading to digital technology applications not aligning closely with actual enterprise needs [30], thereby diminishing their impact on productivity enhancement. Private enterprises tend to be more market-oriented, enabling flexible resource adjustments and optimizations based on actual business needs, allowing digital transformation to more directly enhance labor productivity. Lastly, SOEs are more prone to deficiencies in incentive mechanisms, suppressing employee enthusiasm and initiative, whereas private enterprises typically place more emphasis on performance and incentive mechanisms, allowing digital technologies to better enhance labor productivity [31]. Hence, despite significant resource investment in digital transformation by SOEs, their impact on labor productivity is relatively weaker than that of private enterprises. Therefore, the paper proposes Proposition 1:

#### Proposition 1.

The impact of digital transformation on labor productivity in state-owned enterprises is weaker than in non-state-owned enterprises.

### 1.4 Heterogeneity analysis of industry chain dependency levels

In the process of digital transformation, enterprises require significant resource input, including capital, technology, and human resources. The varying levels of industry chain dependency among enterprises result in different priorities and focus areas in digital transformation, causing the impact of digital transformation on labor productivity to vary based on industry chain dependency levels. Specifically, enterprises with high industry chain dependency often need to manage complex supply chains and multi-party coordination, requiring higher levels of production management. Digital transformation optimizes production management, minimizing downtime and resource waste, thus directly enhancing labor productivity. Secondly, from a market mechanism perspective, enterprises with high industry chain dependency often face more complex impacts on production plans and labor resource allocation due to market demand changes [32]. Digital transformation allows enterprises to more accurately predict market demand and optimize production processes and supply chain management, enabling more flexible production task adjustments to maximize each unit of labor’s production capability, thereby increasing labor productivity. Lastly, enterprises with high industry chain dependency usually need to collaborate closely with multiple upstream and downstream partners. Digital technologies like blockchain and collaborative management platforms can break down information silos, reducing inefficiencies caused by information asymmetry or poor communication. By enhancing transparency and traceability across all links, digital transformation not only optimizes internal labor productivity but also enhances the overall efficiency of the entire industry chain, ensuring that each unit of labor input generates greater value throughout the chain.

#### Proposition 2.

The impact of digital transformation is greater in enterprises with high industry chain dependency than in those with low industry chain dependency.

## 2. Research design

### 2.1 Sample selection and data sources

This study uses a sample of companies listed on the Shanghai and Shenzhen A-share stock markets from 2010 to 2023. The data processing steps are as follows: (1) Financial companies, which are non-manufacturing industries, are excluded from the sample of listed companies. (2) To mitigate the influence of extreme values, the variables are further trimmed at the 1st and 99th percentiles. Missing data is supplemented using interpolation methods. The raw data for this study primarily comes from the CSMAR database and statistical yearbooks. The digitalization indicators are based on the results of text analysis of annual reports from listed companies, with the annual report data sourced from the official websites of the Shenzhen Stock Exchange and the Shanghai Stock Exchange.

### 2.2 Variable and model specification

The following model is specified to study the impact of corporate digital transformation on labor productivity:



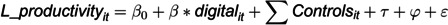



Where:

- ${L\_productivity}_{it}\;$ represents the labor productivity level of company *i* at time *t*;
- ${digital}_{it}\;$ reflects the degree of digital transformation of the company;
- ${Controls}_{it}\;$ is a set of control variables;
- *τ* and *ϕ* represent time and individual fixed effects, respectively;
- ∊ is the random error term.

(1) Dependent Variable: Corporate Labor Productivity. Labor productivity is an indicator used to measure the amount of output produced or services provided by workers within a specific period, serving as an important benchmark for evaluating employee efficiency and economic performance. According to the National Bureau of Statistics, overall labor productivity is the ratio of GDP to the total number of employed persons in the same period, with the calculation formula: Overall Labor Productivity = GDP/Average Annual Employment. At the company level, labor productivity is generally measured as the output created per employee. Drawing on previous research and considering data availability [33], this study adopts the measure of labor productivity as the natural logarithm of the ratio of operating income to the number of employees.
(2) Key Independent Variable: Corporate Digital Transformation. There are several ways to measure corporate digital transformation. Some scholars use binary variables (0–1) derived from annual report information to represent the extent of digital transformation [34]. Other studies measure digitalization by the proportion of intangible assets related to digital transformation in the total intangible assets, as stated in the financial report notes [35]. However, many studies use the frequency of digital technology-related terms in the annual reports of listed companies to represent the degree of digital transformation [36]. This is because frequency analysis offers a quantitative approach that can intuitively reflect the company’s emphasis on and investment in digital technology, while also shedding light on the focus areas of digital transformation.

Thus, this study chooses to use term frequency analysis from the annual reports of listed companies as a proxy for the level of digital transformation. Specifically, the natural logarithm of the number of times digital-related terms appear in the annual report is used to measure the digitalization level. The term library used for text analysis is based on the approach by Wu Fei et al [37]. The digital transformation of enterprises can be divided into two categories: digital technology application and application scenario innovation. The application of digital technology focuses on the adoption and use of underlying technologies such as big data, artificial intelligence, and the Internet of Things, reflecting the ability to introduce technical tools. On the other hand, application scenario innovation emphasizes the deep integration of technology with the actual business of the enterprise, such as new models like smart factories and telemedicine, reflecting the pathways to realizing the value of technology. These correspond to the “technical foundation” and “business restructuring” aspects of digital transformation, covering the entire process from technology implementation to value creation, thus avoiding the limitations of single-dimensional analysis.

The final term library used to represent digital transformation is presented in Table 1.

**Table 1:** Digitalization keyword lexicon.

Level	Category	Keywords
Digital Technology Applications	Big Data	Big Data, Data Collection, Digital Technology, Digital Twin, Data-Driven, Data Services, Data Storage, Data Exchange, Data Processing, Data Mining, Text Mining, Data Visualization, Heterogeneous Data, Virtual Reality, Data Security, Data Lake
	Artificial Intelligence	Robots, Business Intelligence, Image Understanding, Decision Support System, Data Governance, Intelligent Data Analysis, Intelligent Robots, Machine Learning, Deep Learning, Semantic Search, Biometric Recognition, Facial Recognition, Voice Recognition, Identity Verification, Autonomous Driving, Natural Language Processing, Brain-Computer Interface, Metaverse
	Mobile Internet	Internet, Industrial Internet, Mobile Internet, Internet of Things, Information Technology, Information Systems, Blockchain, Digital Currency, Distributed Computing, High-Tech
	Cloud Computing	Cloud, Cloud Platform, Cloud Services, Stream Computing, Graph Computing, In-Memory Computing, Multi-Party Secure Computing, Brain-Inspired Computing, Green Computing, Cognitive Computing, Fusion Architecture, Cyber-Physical Systems
Application Scenarios Innovation	Intelligent, Informational, Digitalized, Networked, Digital Economy, Data Center, Digital Transformation, Databases, Remote, Unmanned, Autonomous Driving, Management Systems, Telemedicine, Data Platforms, Open Platforms, Digital Platforms, Information Networks, Technology Industry, Internet Healthcare, E-Government, E-Commerce Platforms, E-Commerce, New Retail, Mobile Payments, Third-Party Payments, Smart Energy, Connected Networks, Online, Smart Manufacturing, Smart Wearables, Smart Agriculture, Smart Elderly Care, Smart Transportation, Smart Healthcare, Smart Customer Service, Smart Home, Smart Tourism, Smart Environmental Protection, Smart Grid, Smart Marketing, Digital Marketing, Unmanned Retail, Internet Finance, Digital Finance, Fintech, Quantitative Finance, Open Banking, Online Sales, B2B, B2C, C2B, C2C, O2O, OMO

(3) Control Variables:In constructing the econometric model, this study follows a dual principle in selecting control variables: theoretical relevance and comparability with existing literature. The goal is to minimize omitted variable bias and enhance the reliability of the research findings.

Firstly, in terms of firm characteristics, total assets are included in the model as a proxy for firm size, considering that firm size may influence the intensity of digital transformation investment through differences in resource endowments. Firm age is used to capture the potential impact of organizational inertia and accumulated experience on technology adoption [38].

Regarding financial structure, the asset-liability ratio (ALR) reflects the firm’s capital structure, which may affect its financial stability and financing capacity. Since a company’s financial position may influence its investment ability and outcomes in the digital transformation process, it is necessary to control for this variable to isolate its potential effect on productivity. The accounts receivable turnover ratio (ART) measures the efficiency of working capital management, which may moderate the speed of technological iteration, while capital intensity reflects the technological characteristics of the production function [39].

Secondly, from the perspective of corporate governance, the shareholding ratio of the top ten shareholders (Top10) is used to represent ownership concentration, which can influence strategic decision-making. Separation of ownership and control (Sepa_Owner) controls for the possible distortion in digital transformation willingness caused by the divergence between the interests of the actual controller and those of the company. Board size and the proportion of independent directors serve as proxy variables for corporate governance quality. The former affects decision-making efficiency, while the latter reflects the effectiveness of the supervisory mechanism. Both may moderate the process of digital transformation through the mediating role of governance structure.

The main data for this part are derived from corporate annual reports and the CSMAR database, with the specific definitions of variables provided in Table 2.

**Table 2:** Basic definitions of key variables and descriptive statistics.

Variable Type	Variable Name	Variable Symbol	Calculation Method	Observations	Mean	Standard Deviation	Minimum	Maximum
Dependent Variable	Labor Productivity	lnl_productivity	The logarithmic value of labor output per unit of the enterprise	44183	13.845	0.896	3.418	16.502
Core Explanatory Variables	Digital Transformation of the Enterprise	lndigital_sum	Word frequency data	44183	1.278	1.384	0	5.063
	Digital Technology Application	lndigital_tec	Word frequency data	44183	0.693	1.057	0	4.304
	Digital Technology Innovation in Scenarios	lndigital_inn	Word frequency data	44183	1.021	1.225	0	4.522
Control Variables	Asset-liability Ratio	alr	The ratio of total liabilities to total assets at the end of the year	44183	0.423	0.216	0.049	0.972
	Capital Intensity	capital_intensity	Net fixed assets per employee	44183	−1.674	3.273	−4.605	3.351
	Ownership Concentration	top10	The shareholding ratio of the top 10 largest shareholders	44183	58.961	15.9	22.58	94.98
	Firm Age (or Duration of Enterprise)	age	The difference between the current year and the year of establishment	44183	18.443	6.079	5	34
	Separation of Ownership and Control	lnsepa_owner	(First largest shareholder’s shareholding ratio – actual controller’s shareholding ratio)/ first largest shareholder’s shareholding ratio	44183	2.785	3.553	0.405	42.828
	Firm Size	size	Total assets of the enterprise at the end of the year	44183	14.562	51.37	0.3	660
	Accounts Receivable Turnover Ratio	art	Ratio of sales revenue to average accounts receivable	44183	0.258	0.251	0	1.4
	Board Size	board_size	Total number of members in the board of directors	44183	3.804	1.179	2	8
	Proportion of Independent Directors	indep_director	The proportion of independent directors in the board of directors	44183	0.382	0.073	0.25	0.6

Table 2 presents the descriptive statistics for the key variables. As shown in Table 2, the minimum value of labor productivity is 3.418 and the maximum value is 16.502, indicating significant variation in labor productivity across firms. Additionally, the digitalization index for firms has a minimum value of 0 and a maximum value of 5.063, also reflecting considerable variation among firms.

## 3. Empirical analysis

Table 3 presents the results of the baseline regression.*** denotes statistical significance at the 1% level, ** at the 5% level, and * at the 10% level; the same convention applies in the following tables/figures. Column (1) shows the overall effect of corporate digital transformation on labor productivity. The digital transformation of firms is further divided into digital technology application and digital technology scenario innovation, which correspond to columns (2) and (3) of Table 4. It can be seen that the effect of corporate digital transformation on labor productivity is significant at the 0.01 significance level, and the effect is positive. This confirms the basic hypothesis of this study, validating Hypothesis 1.

**Table 3:** Baseline regression results.

	(1)	(2)	(3)
	lnl_productivity	lnl_productivity	lnl_productivity
lndigital_sum	0.022***		
	(0.003)		
lndigital_tec		0.017***	
		(0.004)	
lndigital_inn			0.028***
			(0.003)
alr	0.053***	0.053***	0.054***
	(0.019)	(0.019)	(0.019)
capital_intensity	−0.078***	−0.078***	−0.078***
	(0.001)	(0.001)	(0.001)
top10	0.002***	0.002***	0.002***
	(0.000)	(0.000)	(0.000)
age	0.019**	0.019**	0.020***
	(0.008)	(0.008)	(0.008)
lnsepa_owener	0.002	0.002	0.002
	(0.001)	.0.001	(0.001)
total_assets	0.001***	0.001***	0.001***
	(0.000)	(0.000)	(0.000)
art	−0.308***	−0.308***	−0.307***
	(0.017)	(0.017)	(0.017)
board_size	0.001	0.001	0.001
	(0.003)	(0.003)	(0.003)
indep_director	−0.010	−0.014	−0.010
	(0.051)	(0.051)	(0.051)
Time fixed-effect	Y	Y	Y
Individual fixed-effect	Y	Y	Y
R_sq	0.206	0.205	0.207
Obs.	44183	44183	44183

*** indicates significance at the 0.01 level, ** indicates significance at the 0.05 level, and * indicates significance at the 0.1 level.

**Table 4:** Robustness check by controlling for multidimensional fixed effects and excluding data from the pandemic period.

	(1)	(2)	(3)	(4)
	lnl_productivity	lnl_productivity	lnl_productivity	lnl_productivity
lndigital_sum	0.027***	0.022***	0.030***	0.029***
	(0.003)	(0.002)	(0.004)	(0.004)
_cons	13.810***	13.530***	13.728***	13.828***
	(0.005)	(0.133)	(0.005)	(0.201)
Control Variables	N	Y	N	Y
Time fixed-effect	Y	Y	Y	Y
individual fixed-effect	Y	Y	Y	Y
City fixed-effect	Y	Y	N	N
Industry fixed-effect	Y	Y	N	N
R_sq	0.002	0.206	0.002	0.195
N	44183	44183	27709	27709

*** indicates significance at the 0.01 level, ** indicates significance at the 0.05 level, and * indicates significance at the 0.1 level.

Furthermore, the coefficient of digital technology scenario innovation is greater than the coefficient of digital technology application. The Z-value for this difference is significant at the 0.05 significance level, indicating that, overall, digital technology scenario innovation has a more pronounced effect on labor productivity compared to the mere application of digital technologies.

From a theoretical perspective, digital technology scenario innovation typically involves profound changes to existing business models and processes. By introducing intelligent, informational, and networked solutions, firms can optimize and restructure their business processes, leading to substantial improvements in labor productivity. For example, innovative scenarios such as telemedicine, smart manufacturing, and precision agriculture are not merely the simple addition of technologies. Rather, through the deep integration of technologies, they bring about fundamental transformations in production methods and service models, significantly enhancing efficiency and effectiveness. Therefore, compared to the simple application of digital technologies, digital technology scenario innovation is more likely to facilitate improvements in labor productivity.

### 3.1 Robustness checks

To further enhance the reliability of the regression results, this study conducts the following robustness checks:

#### Controlling for multi-dimensional fixed effects.

This study controls for individual and time-fixed effects, and additionally accounts for industry and city-fixed effects. Different industries and cities may exhibit unique external environments and policy factors that could potentially influence the relationship between corporate digitalization levels and labor productivity. First, the digitalization level and labor productivity across industries may vary significantly. For instance, industries such as information technology and finance tend to have higher levels of digitalization, while traditional sectors like manufacturing and agriculture may exhibit relatively lower levels. Furthermore, labor productivity in different industries may be influenced by industry-specific characteristics and technological advancement. Additionally, factors such as the economic development level, policy environment, and infrastructure in the cities where firms are located may impact both the digitalization process and labor productivity. Firms located in first-tier cities and economically developed regions typically have better digital infrastructure and talent reserves, making it easier for them to achieve digital transformation, thereby enhancing productivity. Therefore, controlling for industry and city fixed effects helps mitigate the potential influence of regional and industry heterogeneity on the regression results. As shown in Columns (1) and (2) of Table 4, after controlling for these multi-dimensional fixed effects, the regression results remain significantly positive.

Excluding Data from the COVID-19 Pandemic Period: The outbreak of the COVID-19 pandemic caused severe disruptions across industries, with many firms facing shutdowns, supply chain interruptions, and demand shrinkage. This led to significant distortions in economic activity. Under such conditions, the digitalization process and labor productivity may experience abnormal fluctuations, deviating from normal economic cycles. To address this, the study excludes data from the pandemic period to conduct a robustness check. The results presented in Columns (3) and (4) of Table 4 show that after excluding the pandemic-period data, the regression results remain significantly positive.

Substituting the Core Explanatory Variable: Following established practices in the literature, this study uses the digital transformation index from the CSMAR database to re-examine the effect of digital transformation on labor productivity [40]. As shown in Columns (5) and (6) of Table 5, after replacing the core explanatory variable, the regression results continue to show a significant positive relationship.

**Table 5:** Robustness check by replacing explanatory variables and controlling for firm strategic behavior.

	(5)	(6)	(7)	(8)
	lnl_productivity	lnl_productivity	lnl_productivity	lnl_productivity
lndigital_sum	0.030***	0.030***	0.027***	0.023***
	0.004	(0.003)	(0.003)	(0.003)
_cons	13.812***	13.488***	13.810***	13.528***
	(0.004)	(0.134)	(0.005)	(0.134)
Control Variables	N	Y	N	Y
Time fixed-effect	Y	Y	Y	Y
Individual fixed-effect	Y	Y	Y	Y
City fixed-effect	N	N	N	N
Industry fixed-effect	N	N	N	N
R_sq	0.002	0.206	0.002	0.1875
N	44183	44183	41520	41520

*** indicates significance at the 0.01 level, ** indicates significance at the 0.05 level, and * indicates significance at the 0.1 level.

Controlling for Strategic Behaviors of Firms: Some firms may have already implemented digital transformation, but did not reflect it in their annual reports. To account for this, this study excludes firms with a constant digitalization level of zero and re-estimates the regression. As shown in Columns (7) and (8) of Table 6, after excluding firms with strategic behaviors, the regression results remain robust and significantly positive.

**Table 6:** Handling of endogeneity issues.

	(1)	(2)	(3)
	lnl_productivity	lndigital_sum	lnl_productivity
lndigital_sum	0.022***		0.328***
	(0.003)		(0.189)
IV		0.011***	
		(0.002)	
IMR	4.801***		
	(0.276)		
_cons	13.018***		
	(0.136)		
Control Variables	Y	Y	Y
Time fixed-effect	Y	Y	Y
Individual fixed-effect	Y	Y	Y
N	44183	44183	44183
Kleibergen-Paap rk LM statistic		33.650***	
Kleibergen-Paap Wald rk F statistic		33.840	

*** indicates significance at the 0.01 level, ** indicates significance at the 0.05 level, and * indicates significance at the 0.1 level.

### 3.2 Addressing endogeneity issues

This study first applies the Heckman two-step procedure to address potential endogeneity issues. The Heckman two-step method is primarily used to correct for sample selection bias, and it resolves potential endogeneity through the following two steps:

Step 1: Estimation of the Probability of Digital Transformation. In the first step, a binary variable indicating whether a firm engages in digital transformation is generated. The probability of a firm opting to enhance its digitalization level is estimated using a Probit model. This model helps capture the factors influencing a firm’s decision to adopt digital technologies and allows for the construction of an appropriate correction term.

Step 2: Inclusion of the Inverse Mills Ratio. In the second step, the Inverse Mills Ratio (IMR) derived from the first step is introduced into the main regression equation. This step ensures that consistent and unbiased estimates are obtained even in the presence of sample selection bias. By incorporating the Inverse Mills Ratio, unobserved factors that simultaneously affect both the firm’s digitalization decision and labor productivity can be controlled. This allows for a more accurate estimation of the true impact of digitalization on labor productivity, ensuring that other influencing factors are not incorrectly attributed to the effect of digitalization.

The specific results, presented in Column (1) of Table 6, show that after correcting for sample selection bias, the regression results remain significantly positive, reinforcing the robustness of the findings.

The digitalization of enterprises can potentially enhance labor productivity. However, enterprises with high labor productivity also have more resources and motivation to undergo digital transformation, which may lead to a bidirectional causality issue. Furthermore, if key variables influencing labor productivity and enterprise digitalization (such as management level, innovation capability, and employee quality) are omitted from the regression model, these omissions could lead to biased estimation results. Therefore, this paper further employs instrumental variables to control for potential endogeneity issues.

Drawing upon the approaches of relevant scholars, this study constructs instrumental variables using the product of the Big Data Comprehensive Experimental Zone and the city’s import-export volume [41]. This is because the establishment of a national Big Data Comprehensive Experimental Zone involves significant resource allocation, typically accompanied by substantial investments in information infrastructure and the promotion of digital applications, directly facilitating the digitalization process of enterprises. Simultaneously, the establishment of such zones is usually based on macroeconomic policies and regional development strategies, relatively independent of the operational conditions and decisions of individual enterprises, thus meeting the requirements of relevance and exogeneity for instrumental variables. An increase in total import and export volume generally reflects the openness and information flow within a city, which promotes the digital development of enterprises. Moreover, the import and export volume is mainly influenced by the overall economic environment of the city and international trade policies, having no direct link to the specific operational decisions of individual enterprises, thereby satisfying the exogeneity condition for instrumental variables. The Big Data Comprehensive Experimental Zone policy primarily reflects the governmental push for digitalization, while the total import and export volume reflects the vibrancy of urban economic activities. The product form integrates the impacts of both policy and economic activities, effectively reflecting the comprehensive digital environment of enterprises, enhancing the correlation between the instrumental variable and enterprise digitalization, and improving the explanatory power and validity of the instrumental variable, thus providing a more representative instrumental variable. The regression results using instrumental variables are shown in columns (2) and (3) of Table 7. Column (2) presents the first-stage regression results for the instrumental variable, with the regression coefficient of the instrumental variable being significant at the 0.01 significance level. Column (3) provides the second-stage regression results, indicating that the regression results remain significantly positive after using the instrumental variable. Additionally, the P-value of the Kleibergen-Paap rk LM statistic is 0.00, indicating that there is no issue of under-identification with the instrumental variable. For testing weak instrumental variables, the Kleibergen-Paap rk Wald F statistic exceeds the critical value of 16.38 at the 10% level of the Stock-Yogo weak identification test, suggesting that the instrumental variables selected in this study are valid.

**Table 7:** The mediating effect of production management.

	(1)	(2)	(3)	(4)	(5)	(6)
	inventory	lnl_productivity	inventory	lnl_productivity	inventory	lnl_productivity
Inventory		0.034***		0.001***		0.001***
		(0.002)		(0.000)		(0.000)
lndigital_inn	0.952***	0.029***				
	(0.281)	(0.003)				
lndigital_tec			1.065***	0.015***		
			(0.313)	(0.003)		
lndigital_sum					0.934***	0.022***
					(0.258)	(0.003)
_cons	2.902	13.254***	3.098	13.313***	2.932	13.304***
	(7.709)	(0.084)	(7.707)	(0.084)	(7.708)	(0.084)
Control Variables	Y	Y	Y	Y	Y	Y
Time fixed-effect	Y	Y	Y	Y	Y	Y
individual fixed-effect	Y	Y	Y	Y	Y	Y
Sobel	3.573***		3.570***		3.801***	
bootstrap	[0.0002,0.001]		[0.0002,0.002]		[0.0002, 0.001]	
N	44183	44183	44183	44183	44183	44183
R-sq	0.006	0.336	0.006	0.335	0.006	0.336

*** indicates significance at the 0.01 level, ** indicates significance at the 0.05 level, and * indicates significance at the 0.1 level.

### 3.3 Mechanism examination enterprise digitalization and production management optimization

Digitalization assists enterprises in achieving lean production, which implies “Just-In-Time” (JIT) manufacturing and production on demand [42]. This indicates that, to fully leverage the advantages of digital technology and enhance operational efficiency, enterprises will coordinate the supply and demand sides to minimize inventory, producing and procuring only when necessary. At the same time, by optimizing production processes and reducing production cycle times, products can reach the market faster, directly leading to a decrease in average inventory levels, as reflected in inventory turnover rates [43]. Therefore, this paper uses inventory turnover rate as an indicator of production management optimization.

The specific regression results are shown in Table 7. Columns (1), (3), and (5) of Table 7 present the first-stage regression results of the mediation effect. It can be seen that the regression coefficients of digital technology scenario innovation, digital technology application, and enterprise digitalization are significant, and they have a positive impact on the enterprise’s inventory turnover rate. Columns (2), (4), and (6) provide the second-stage regression results, where the coefficients of the mediating variable and the core explanatory variable are significantly positive, indicating that enterprise digitalization can enhance labor productivity through the optimization path of production management. On this basis, the paper further conducted a Sobel test, finding that the Z-value statistics are significant at the 1% level. Additionally, a Bootstrap (1000 times) sampling test was conducted, showing that the 95% confidence intervals for the mediating effect do not include zero. In summary, Hypothesis 2 of this paper is validated.

#### Digitalization and the improvement of corporate internal control.

This paper uses the DIBO Internal Control Index to represent the quality of corporate internal control. The DIBO Internal Control Index is the result of a systematic evaluation based on the five key components of internal control: control environment, risk assessment, control activities, information and communication, and monitoring. These components are core elements of the COSO (Committee of Sponsoring Organizations of the Treadway Commission) Internal Control Framework, which is widely recognized internationally and has a solid theoretical foundation. Furthermore, the DIBO Internal Control Index provides a comprehensive analysis and quantification of publicly disclosed internal control information, covering the main aspects of both accounting and management controls, ensuring objectivity and consistency in the evaluation results [44]. The index employs a combination of quantitative and qualitative assessment methods, taking into account not only the design and implementation of internal control systems but also the supervision and feedback mechanisms in actual operations, making its evaluation results more comprehensive and representative [45].

The regression results are shown in Table 8. Columns (1), (3), and (5) present the results of the first stage of the mediation effect regression, revealing that digital technology-driven innovation, digital technology applications, and corporate digitalization have a significantly positive impact on the improvement of internal control. Columns (2), (4), and (6) display the results of the second stage of regression, where the coefficients of the mediating variables and core explanatory variables are significantly positive, indicating that corporate digitalization can enhance labor productivity through the improvement of internal control. On this basis, the paper further conducts a Sobel test, which shows that the Z-value statistic is significant at the 1% level. Additionally, a Bootstrap (1000 iterations) sampling test is performed, revealing that the confidence interval of the mediating effect at a 95% confidence level does not include 0. In conclusion, Hypothesis 3 is validated.

**Table 8:** The mediating effect of internal control.

	(1)	(2)	(3)	(4)	(5)	(6)
	inter_con	lnl_productivity	inter_con	lnl_productivity	inter_con	lnl_productivity
inter_con		0.011***		0.011***		0.011***
		(0.001)		(0.001)		(0.001)
lndigital_inn	0.143***	0.028***				
	(0.016)	(0.003)				
lndigital_tec			0.173***	0.014***		
			(0.017)	(0.003)		
lndigital_sum					0.162***	0.021***
					(0.014)	(0.003)
_cons	8.642***	13.205***	8.664***	13.218***	8.628***	13.211***
	(0.429)	(0.084)	(0.429)	(0.084)	(0.429)	(0.084)
Control Variables	Y	Y	Y	Y	Y	Y
Time fixed-effect	Y	Y	Y	Y	Y	Y
Individual fixed-effect	Y	Y	Y	Y	Y	Y
Sobel	7.979***		8.089***		8.737***	
bootstrap	[0.001,0.002]		[0.001, 0.002]		[0.001,0.002]	
N	44183	44183	44183	44183	44183	44183
R-sq	0.284	0.333	0.283	0.335	0.284	0.334

*** indicates significance at the 0.01 level, ** indicates significance at the 0.05 level, and * indicates significance at the 0.1 level.

### 3.4 Heterogeneity test: Analysis based on ownership structure

A regression analysis is conducted by distinguishing between state-owned enterprises (SOEs) and non-state-owned enterprises (non-SOEs). The detailed results are shown in Table 9. Columns (1), (3), and (5), as well as columns (2), (4), and (6), present the regression results for the impact of digital technology usage, application scenario innovation, and corporate digitalization on labor productivity for SOEs and non-SOEs, respectively.

**Table 9:** Results of heterogeneity analysis based on property rights.

	(1)	(2)	(3)	(4)	(5)	(6)
	lnl_productivity	lnl_productivity	lnl_productivity	lnl_productivity	lnl_productivity	lnl_productivity
lndigital_inn	0.014**	0.030***				
	(0.006)	(0.004)				
lndigital_tec			−0.001	0.019***		
			(0.006)	(0.004)		
lndigital_sum					0.009*	0.024***
					(0.005)	(0.003)
_cons	13.361***	13.300***	13.370***	13.318***	13.365***	13.309***
	(0.138)	(0.111)	(0.138)	(0.111)	(0.138)	(0.111)
Control Variables	Y	Y	Y	Y	Y	Y
Time fixed-effect	Y	Y	Y	Y	Y	Y
Individual fixed-effect	Y	Y	Y	Y	Y	Y
N	14571	29612	14571	29612	14571	29612
R-sq	0.195	0.222	0.195	0.221	0.195	0.221

*** indicates significance at the 0.01 level, ** indicates significance at the 0.05 level, and * indicates significance at the 0.1 level.

It is evident that for the SOE sample, the innovation of digital technology application scenarios has a significantly positive impact at the 0.05 significance level, but the effect is only 50% of that observed for non-SOEs. For SOEs, the coefficient of digital technology application is not significant, while the overall effect of corporate digitalization is significant at the 0.1 significance level, with the effect level being notably lower than that of non-SOEs. Moreover, for SOEs, the coefficient of labor productivity concerning digital technology application is not significant, while the impact of digital technology scenario innovation on labor productivity is significant at the 0.1 significance level, though the coefficient is lower than that of the non-SOE sample.

This phenomenon indicates a clear difference in the way and results of digital transformation between SOEs and private enterprises. This can be explained by differences in organizational structures and market environments. For state-owned enterprises, due to their relatively rigid organizational structure and complex decision-making processes, the mere application of digital technologies is unlikely to rapidly translate into actual productivity improvements. In contrast, scenario innovation typically involves redesigning and optimizing business processes, rather than just introducing new technological tools. Such comprehensive reforms can address redundancy and inefficiencies that exist within SOEs, leading to a more significant increase in labor productivity. Additionally, SOEs often have strong resource integration capabilities and policy support, which gives them an advantage in applying innovative digital scenarios.

In contrast, private enterprises exhibit higher flexibility and market sensitivity, enabling them to quickly adapt to and adopt new technologies. As a result, both the direct application of digital technologies and the innovation of application scenarios can more quickly translate into actual productivity gains. This flexibility and adaptability allow private enterprises to leverage various digital technologies more efficiently during their digital transformation, thereby achieving a more comprehensive improvement in labor productivity. Therefore, Hypothesis 1 is validated.

#### Heterogeneity analysis based on supply chain dependency.

Drawing on the approach used by relevant scholars, this paper adopts the ratio of the transaction amount with the main supplier or main customer to the total annual transaction amount as the supply chain dependency indicator [46]. The sample is then divided into low dependency and high dependency groups using the median of this indicator. The resulting regression results are presented in Table 10. Columns (1), (3), and (5) show the regression results for the low dependency group, while columns (2), (4), and (6) present the results for the high dependency group.

**Table 10:** Heterogeneity analysis based on the level of industry chain dependence.

	(1)	(2)	(3)	(4)	(5)	(6)
	lnl_productivity	lnl_productivity	lnl_productivity	lnl_productivity	lnl_productivity	lnl_productivity
lndigital_inn	0.018***	0.044***				
	(0.004)	(0.005)				
lndigital_tec			−0.001	0.039***		
			(0.004)	(0.005)		
lndigital_sum					0.012***	0.037***
					(0.004)	(0.004)
_cons	13.295***	13.283***	13.306***	13.302***	13.297***	13.289***
	(0.106)	(0.139)	(0.106)	(0.139)	(0.106)	(0.139)
Control Variables	Y	Y	Y	Y	Y	Y
Time fixed-effect	Y	Y	Y	Y	Y	Y
individual fixed-effect	Y	Y	Y	Y	Y	Y
N	22105	22078	22105	22078	22105	22078
R-sq	0.166	0.267	0.165	0.266	0.166	0.267

*** indicates significance at the 0.01 level, ** indicates significance at the 0.05 level, and * indicates significance at the 0.1 level.

It can be observed that the impact of corporate digitalization and digital scenario innovation on labor productivity remains significant across both groups. However, for the low dependency group, the effect of digital technology application on labor productivity is not significant. This may be because companies with low supply chain dependency typically have simpler business models, often centered around a single product or service. The complex application of digital technologies in these business contexts may not fully unleash their potential, resulting in a limited effect on overall labor productivity improvement. Therefore, Hypothesis 2 is validated.

## 4. Further discussion: The moderating role of industry characteristics

In the previous sections, we explored the relationship between digital transformation and labor productivity from the perspective of internal business process restructuring within firms. However, the industry in which a firm operates, as an external environment essential for its survival and development, inevitably has a profound impact on corporate behavior. Different industries exhibit significant variations in market structure, competitive dynamics, technological levels, and other factors, which can lead to diverse patterns and extents of digitalization’s impact on labor productivity across industries.

### 4.1 The moderating role of industry monopoly level

This paper further considers the potential moderating effect of industry monopoly levels. In highly competitive industries, firms face greater pressure to enhance efficiency and control costs, leading them to focus their resources on digitalization projects that are directly related to their core business in order to rapidly improve production efficiency. In these industries, digital transformation becomes a crucial means for firms to maintain their competitive advantage and improve productivity. On the other hand, in industries with a higher level of market monopoly, firms may face less market competition pressure, leading to a relatively weaker motivation to proactively pursue digital transformation. These firms may place more emphasis on the long-term impact of digital transformation on maintaining their competitive edge and monopolistic position, leading to a more dispersed resource allocation and potentially weaker direct effects on labor productivity improvement.

Thus, the degree of industry monopoly can lead to differences in firms’ enthusiasm for digital transformation, which in turn affects the impact of digitalization on labor productivity. This study uses the Herfindahl-Hirschman Index (HHI) to represent the monopoly level of the industry in which a firm operates, and the specific results are shown in columns (1), (2), and (3) of Table 11.

**Table 11:** Moderating effect of industry-wide digitalization level.

	(1)	(2)	(3)	(4)	(5)	(6)
	lnl_productivity	lnl_productivity	lnl_productivity	lnl_productivity	lnl_productivity	lnl_productivity
lndigital*adj_var	−0.022***	−0.020*	−0.019***	0.0195***	0.0003***	0.0005***
	(0.008)	(0.011)	(0.007)	(0.003)	(0.000)	(0.000)
lndigital_inn	0.034***			0.001***		
	(0.003)			(0.000)		
lndigital_tec		0.019***			0.009**	
		(0.004)			(0.004)	
lndigital_sum			0.026***			0.014***
			(0.003)			(0.003)
_cons	13.298***	13.314***	13.304***	13.291***	13.311***	13.297***
	(0.084)	(0.084)	(0.084)	(0.084)	(0.084)	(0.084)
Control Variables	Y	Y	Y	Y	Y	Y
Time fixed-effect	Y	Y	Y	Y	Y	Y
individual fixed-effect	Y	Y	Y	Y	Y	Y
N	44183	44183	44183	44183	44183	44183
R-sq	0.333	0.331	0.332	0.244	0.242	0.243

*** indicates significance at the 0.01 level, ** indicates significance at the 0.05 level, and * indicates significance at the 0.1 level.

The first row of Table 11 presents the regression results for the moderating effect. It is evident that the coefficients for the monopoly moderating effect are negative and significant, which is in line with expectations. The coefficients for digital technology scenario innovation and overall digitalization level are significant at the 0.01 significance level, while the moderating effect of monopoly on digital technology application is the least pronounced, being only significant at the 0.1 significance level. This may be because firms in monopolistic industries tend to have already established a relatively stable competitive advantage and face lower market pressure. As a result, their internal motivation and sense of urgency are insufficient, which, in turn, weakens their enthusiasm for comprehensively advancing digital transformation.

For digital technology scenario innovation, this approach involves broader organizational changes and business process reengineering, which are more likely to be constrained and hindered by the market structure in monopolistic industries. Consequently, firms in these industries may need to invest more effort to achieve digital transformation, which makes them more susceptible to the impact of insufficient internal motivation. In contrast, digital technology applications are more focused on the technical level and have less of an impact on existing operational practices, meaning they are less influenced by the level of industry monopoly.

### 4.2 The moderating role of industry’s overall digitalization level

Digital transformation exhibits network effects, meaning that the overall digitalization level of the industry may influence the effectiveness of digital transformation within firms. First, the improvement in industry-level digitalization is often accompanied by the diffusion of knowledge and technology. In a highly digitized industry environment, there is more frequent technological exchange and information sharing between firms, which helps individual companies access advanced digital technologies and management experiences, thus accelerating their own digitalization process. Moreover, the higher the level of digitalization in an industry, the more pronounced the industry’s collaborative effect across the value chain. In highly digitized industries, collaboration between upstream and downstream firms becomes more seamless, and information sharing and resource integration are more efficient [47]. This collaborative effect enables individual firms to optimize supply chain management, enhance production process efficiency, and reduce operational costs, thereby indirectly improving labor productivity.

Thus, this study calculates the average digital transformation level within a specific period for firms belonging to a given industry to represent the overall digital transformation level of that industry (IDT). The specific regression results are shown in columns (4), (5), and (6) of Table 11.

The first row of Table 11 presents the regression results for the moderating effect. It is evident that the moderating effect of the industry’s digital transformation level is positive, and the coefficients are significant at the 0.01 significance level, indicating that when the overall digital transformation level of the industry is high, the effect of digital transformation on labor productivity improvement within firms becomes more pronounced.

Based on the perspective of business process reengineering, this study explores the effect of digital transformation on labor productivity in the real economy and provides a detailed discussion and verification through theoretical analysis and empirical research. Using data from Shanghai and Shenzhen A-share listed companies between 2010 and 2023, and constructing appropriate econometric models with multi-dimensional robustness tests, the following key conclusions were drawn:

The overall effect of digital transformation on labor productivity is significant. The impact of digital technology scenario innovation on labor productivity is more pronounced than that of merely applying digital technologies.

Through the mediation effect analysis of digital transformation, it was found that digital transformation improves labor productivity by optimizing production management processes and enhancing internal control mechanisms. Digitalization drives enterprises to adopt lean production methods, reduce waste, and increase production efficiency; at the same time, a well-developed internal control system provides more accurate financial and performance data, assisting enterprises in better decision-making and management.

There are differences in the effects of digital transformation on labor productivity across enterprises with different ownership structures. State-owned enterprises, due to their relatively rigid organizational structure, show a lower impact of digital transformation, while non-state-owned enterprises, with greater flexibility and adaptability, are able to more efficiently leverage digital technologies, leading to a significant improvement in labor productivity. Enterprises with high industry chain dependence experience a more pronounced improvement in labor productivity after digital transformation.

The degree of industry monopoly has a significant moderating effect on the outcomes of digital transformation. In highly competitive industries, the effect of digital transformation is more pronounced, and when the overall industry level of digital transformation is higher, the impact of digital transformation on improving labor productivity becomes even more evident.

## Supporting information

S1 Data XXX. (XLSX)
